# A machine learning heuristic to identify biologically relevant and minimal biomarker panels from omics data

**DOI:** 10.1186/1471-2164-16-S1-S2

**Published:** 2015-01-15

**Authors:** Anna L Swan, Dov J Stekel, Charlie Hodgman, David Allaway, Mohammed H Alqahtani, Ali Mobasheri, Jaume Bacardit

**Affiliations:** 1 School of Biosciences, Faculty of Science, University of Nottingham, Sutton Bonington Campus, Leicestershire, LE12 5RD, United Kingdom; 2 The D-BOARD European Consortium for Biomarker Discovery, The Universities of Surrey, Nottingham and Newcastle, United Kingdom; 3 School of Veterinary Medicine, Faculty of Health and Medical Sciences, University of Surrey, Duke of Kent Building, Guildford, Surrey, GU2 7XH, United Kingdom; 4 WALTHAM® Centre for Pet Nutrition, Waltham-on-the-Wolds, Melton Mowbray, Leicestershire, LE14 4RT, United Kingdom; 5 Center of Excellence in Genomic Medicine Research (CEGMR), King AbdulAziz University, Jeddah, 21589, Kingdom of Saudi Arabia; 6 Arthritis Research UK Centre for Sport, Exercise, and Osteoarthritis, Arthritis Research UK Pain Centre, Medical Research Council-Arthritis Research UK Centre for Musculoskeletal Ageing Research, Faculty of Medicine and Health Sciences, University of Nottingham, University Park, Nottingham, NG7 2RD, United Kingdom; 7 The Interdisciplinary Computing and Complex BioSystems (ICOS) research group, School of Computing Science, Newcastle University, Claremont Tower, Newcastle-upon-Tyne, NE1 7RU, United Kingdom

## Abstract

**Background:**

Investigations into novel biomarkers using omics techniques generate large amounts of data. Due to their size and numbers of attributes, these data are suitable for analysis with machine learning methods. A key component of typical machine learning pipelines for omics data is feature selection, which is used to reduce the raw high-dimensional data into a tractable number of features. Feature selection needs to balance the objective of using as few features as possible, while maintaining high predictive power. This balance is crucial when the goal of data analysis is the identification of highly accurate but small panels of biomarkers with potential clinical utility. In this paper we propose a heuristic for the selection of very small feature subsets, via an iterative feature elimination process that is guided by rule-based machine learning, called RGIFE (Rule-guided Iterative Feature Elimination). We use this heuristic to identify putative biomarkers of osteoarthritis (OA), articular cartilage degradation and synovial inflammation, using both proteomic and transcriptomic datasets.

**Results and discussion:**

Our RGIFE heuristic increased the classification accuracies achieved for all datasets when no feature selection is used, and performed well in a comparison with other feature selection methods. Using this method the datasets were reduced to a smaller number of genes or proteins, including those known to be relevant to OA, cartilage degradation and joint inflammation. The results have shown the RGIFE feature reduction method to be suitable for analysing both proteomic and transcriptomics data. Methods that generate large ‘omics’ datasets are increasingly being used in the area of rheumatology.

**Conclusions:**

Feature reduction methods are advantageous for the analysis of omics data in the field of rheumatology, as the applications of such techniques are likely to result in improvements in diagnosis, treatment and drug discovery.

## Background

The 'omics' (genomics, epigenomics, transcriptomics, proteomics, metabolomics and lipidomics) are making significant contributions to the study of chronic diseases, especially the identification of novel biomarkers. A biomarker is defined as a characteristic that may be objectively measured and evaluated as an indicator of normal biologic processes, pathogenic processes, or pharmacologic responses to a therapeutic intervention [1]. Biomarkers are actively investigated in the areas of clinical rheumatology, orthopaedics and sports medicine. Osteoarthritis (OA) is a degenerative joint disease that affects the entire joint structure [2]. It is characterised by progressive degeneration of cartilage, menisci, ligaments and subchondral bone [3,4]. Synovial inflammation (synovitis) is a major contributor to disease progression [5-7] and is responsible for the increased production of catabolic and pro-inflammatory mediators that alter the balance of cartilage matrix degradation and repair, leading to excess production of the proteolytic enzymes responsible for cartilage breakdown [6]. OA is currently diagnosed by radiography, once clinical signs of pain and loss of mobility have already appeared, and therefore biomarkers that could identify early signs of OA would significantly aid in diagnosis [8]. Current research is aimed at identifying panels of clinically useful biochemical and imaging markers into single diagnostic algorithms that can be used for diagnostic and prognostic applications and for testing the efficacy of new drugs [9]. Applying ‘omics’ results in the generation of large datasets that are suitable for bioinformatic analysis using machine learning, to extract important information [10].

Bioinformatics tools play an important role in the analysis of data from omics technologies, such as microarrays, next generation sequencing and mass spectrometry (MS), and as a result a wide range of methods have been developed [11,12]. Such methods include supervised machine learning (ML) techniques, which are used to build classification models. Models are used to automatically label samples of unknown class by using a training set of known labelled samples. There are many different types of ML methods, some of which can be used to identify putative biomarkers from data by observing the attributes (genes or proteins) used to build the models. Rule-based methods are an example of this, as it is possible to read the rules generated to form the model [13].

**Figure 1:**
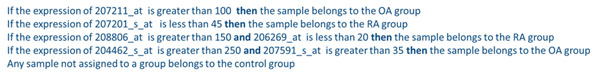
Example of a rule set generated by BioHEL. Rule sets are generated by BioHEL to classify samples. The combination of rules in the rules sets are used to assign samples to their respective treatment groups. Each rule contains one or more gene and an expression value which each gene should either be above or below, depending on the rule. At the end of each line is the group to which each rule relates. For example, the 1st rule of the rule set shown classifies all samples as belonging to the OA class if the value of the gene attribute 207211_at is greater than 100.

BioHEL is a rule based machine learning method which has been used for sample classification in highly dimensional datasets because of its fine-grained embedded feature selection [14]. It has been successfully applied to accurately classify many different types of biological data [15-18]. Rule-based methods construct rule sets that contain at least one rule for each sample group, based on the values associated with the attributes, for example the expression value of the genes. An example of a rule set is shown in Figure 1. ML can also be used to identify possible biomarkers in the form of feature selection (FS), a method of data reduction. FS techniques identify a subset of attributes, for example genes or proteins, which could be used to build a more successful model, compared to using the whole dataset.

Supervised FS methods analyse data with known class labels, with the aim to remove irrelevant or redundant features. Using FS techniques can improve model performance, increase classification accuracy, and provide a clearer understanding of attributes, which are useful for generating an effective classification model. Their key challenge is to avoid a loss of information by dropping features that contain crucial information for the data being analysed. The application of FS methods is particularly relevant for transcriptomic datasets that often have very large numbers of attributes, in relation to the number of samples. There are three main forms of feature selection: filter, wrapper and embedded methods [19]. Filter methods, such as correlation-based feature selection [20], are used to estimate the effectiveness of a reduction in attributes. Wrapper methods, including Genetic Algorithms [21], use classifiers to determine if the subset of attributes gives successful classifications. The third method, embedded, includes feature reduction within the process of classification and uses such machine learning techniques as Naïve Bayes [22] and Support Vector Machines (SVM) [23,24].

The aim of this study was to develop an FS heuristic specialized on identifying very reduced sets of variables built on top of the BioHEL rule-based ML method, in order to increase the accuracy of classification models and to identify putative biomarkers. This heuristic performs an iterative feature elimination process that is guided (in choosing which features to eliminate first) by an analysis of the rule sets generated by BioHEL. This guiding process avoids numerous iterations of blind trial-and-error attempts at removing features from the dataset, and quickly finds, in most datasets, very reduced subsets of features. We call this method RGIFE (Rule-guided Iterative Feature Elimination).

Other work in this area has involved iterative processes for feature reduction, however these have been based on different methods [23]. One example of such a process for feature reduction is Support Vector Machines Recursive Feature Elimination (SVM RFE), which is a form of backward feature elimination. SVM RFE has three main steps. Firstly it trains the SVM classifier, followed by computing a specific ranking criterion for all features. This criterion is based on the weights of a linear SVM. The features are ranked based on this value and the feature(s) with the smallest ranking criterion are removed. These steps are then repeated in an iterative process, resulting in a reduced number of features.

The RGIFE method is tested on several proteomics and transcriptomics OA datasets to demonstrate its suitability for multiple data types. Its performance is compared to several combinations of feature selection and classification algorithms. The results show that RGIFE improves BioHEL’s performance in all datasets, is able to identify very reduced sets of variables in most of them and shows competitive performance to other methods from the literature.

## Methods

### Proteomics and transcriptomics datasets

A proteomics dataset was selected, which had been previously analysed by BioHEL to identify putative biomarkers [25]. The proteomics dataset analysed here consisted of 23 samples analysed by mass spectrometry to identify the proteins present in each sample [26]. The dataset included six samples treated with the pro-inflammatory cytokine interleukin-1 beta (IL-1β), to simulate OA in culture, and five samples treated with IL-1β followed by carprofen, which is a non-steroidal anti-inflammatory drug (NSAID) used to treat OA. Also included in the study were six control samples and six samples treated with only carprofen. Mascot was applied, using the Uniprot database, to determine proteins present in the samples. From this two datasets were generated. The first included an emPAI quantitation value assigned to each protein present and alternatively, the second provided each protein with a ProteinProphet probability [27,28]. This probability is a measure of how likely it is each protein is present in a sample.

To assess the method developed on transcriptomics data, datasets were identified from ArrayExpress [21,23-29] and NCBI GEO [30] by searching for the term ‘Osteoarthritis’. Of the datasets that this search term returned, those that had more than five samples per group (disease or treatment group) were analysed. This resulted in five datasets (Table 1) that vary in size, but which all contain many more attributes than the proteomics dataset. The sample numbers also vary between 25 samples over 5 classes to 48 samples over 3 classes. These datasets were classified using BioHEL and other machine learning methods, as the canine proteomic dataset was, reported in a recent paper by Swan et al., [25].

**Table 1:** Descriptions of datasets analysed, including both proteomic and transcriptomic. Those prefixed 'GSE' were from NCBI GEO and those prefixed 'E-GEOD' were from ArrayExpress.

Dataset	No. of samples	No. of genes	No. of classes	Description
**Proteomics datasets:**				

**emPAI canine**	23	178	4	Articular cartilage dataset treated with IL-1β to stimulate inflammation. Some samples were also treated with carprofen, a non-steroidal anti-inflammatory drug. Other samples were treated with carprofen only or nothing, as a control. The emPAI dataset includes emPAI label-free quantitation to compare protein quantities across samples, for the proteins with Mascot scores above 30. The ProteinProphet dataset includes a probability for each protein identified in each sample indicating how likely it is to be present in the sample.
	
**ProteinProphet canine**	23	1322	4	

**Transcriptomics datasets:**				

**GSE36700**	25	54675	5	Comparison between gene expression in synovial biopsies from patients with OA, RA, Systemic Lupus Erythematosus (SLE), seronegative arthritis (SA), and microcrystalline arthritis (MIC) [48]

**GSE3698**	48	17048	3	Comparison between OA, RA & Pigmented villonodular synovitis (VS), a rare group of lesions with morphological features suggesting an inflammatory as well as a neoplastic nature. All three diseases result in a progressive destruction of affected joints and remain a diagnostic difficulty because of nonspecific symptoms. Tissue samples obtained from knee surgery [49]

**E-GEOD-12021**	31	22284	3	Gene expression variances were tested in synovial membrane samples of RA patients, OA patients, and normal controls [50]

**E-GEOD-29746**	31	44397	3	Comparison of gene expression between two pathological groups of human synovial fibroblasts (SF) from RA and OA synovial tissues with normal SF from healthy individuals [51]

**E-GEOD-27390**	19	54675	2	Gene expression profiling of bone marrow-derived mononuclear cells from patients with RA vs. OA [52]

All datasets were partitioned into training and test sets following the leave-one-out cross-validation methodology.

### The Rule-guided Iterative Feature Elimination heuristic

With the goal of identifying very reduced and highly accurate sets of variables we propose a feature selection heuristic built on top of the BioHEL rule-based machine learning method and based on the iterative feature elimination (IFE) principle. The basic IFE method would pick an attribute, remove it from the dataset and train a classifier (a rule set) with the remaining attributes. If the prediction capacity of the new model is equal or better than with the whole set of attributes (or the previous model), the attribute is eliminated. Otherwise it is inserted back into the dataset. The basic IFE process becomes extremely computationally demanding in high dimensional datasets, such as the case of omics data. Therefore, in order to make such IFE heuristic feasible it needs to be improved in several directions.

The main change is that, rather than picking attributes to be removed at random, we will pick them based on their relevance, estimated from the rule sets generated by BioHEL from the whole set. Then, the first attribute(s) to be picked for elimination are those at the bottom of BioHEL’s ranking.

The second change is remove attributes in blocks, rather than one by one. At the start of the heuristic the block size is set up to a very large number, 25% of the problem’s attributes. If a block cannot be removed because it would decrease the prediction capacity of the classification model then the next block (following the ranking of attributes) is tested. The block size is reduced to be 25% of the previous block size whenever (a) all attributes have been tested or (b) five consecutive unsuccessful trials have been attempted.

As a final change the acceptance criteria of a trial is relaxed in certain scenarios: whenever five consecutive unsuccessful trials have been performed, before reducing the block size the heuristic checks if one of these trials suffered an accuracy drop corresponding to just one sample. If so, this trial is accepted.

**Figure 2:**
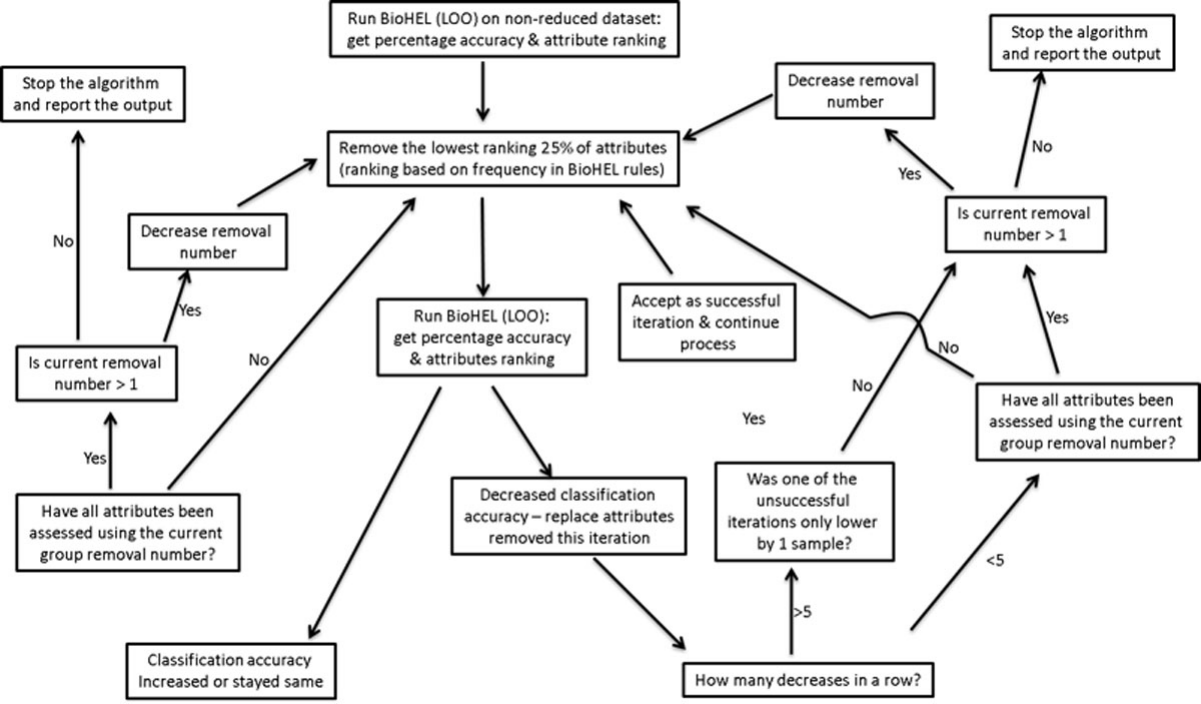
Workflow of the RGIFE heuristic, where, each iteration, genes are removed and only returned if their removal lowers the classification accuracy. (Leave-one-out cross validation was used to assess the classification abilities of the models built).

The iterative process then finishes when the attributes are being removed one at a time and either the removal of all attributes left in the dataset had been tested or five iterations in a row resulted in a drop in the percentage classification accuracy equivalent to more than one sample. The overall workflow of the heuristic is represented in Figure 2.

### Experimental design

For the experiments of this paper our aim is to show that the RGIFE heuristic is both able to find feature subsets that are both (a) small and (b) highly accurate. To this aim we have designed a series of experiments that firstly compares the heuristic with other FS methods, and then compares the accuracy obtained by BioHEL with the reduced feature subsets with other machine learning methods.

The feature selection techniques used were Correlation-based (CFS), SVM RFE, Random Forest, Naïve Bayes and Chi Squared, some of which are feature selection methods based on the machine learning methods used. All methods used were those implemented in the machine learning software, WEKA [31]. For those methods that ranked their selected attributes, rather than identifying a small selection of attributes, the top 10 features were used. The machine leaning methods included in the comparison are Naïve Bayes, Support Vector Machines (SVM), k-nearest neighbour (IBk), JRip (rule-based), J48 and Random Forest (RF), also using WEKA implementations [31].

For all classifications performed, the true positive rate (TPR/sensitivity) and true negative rate (TNR/specificity) were calculated. The TPR is the measure of the proportion of correctly classified samples and multiplying it by 100 gives the percentage classification accuracy. TPR is calculated by:(1)

In contrast, the TNR is a measure of the proportion of negatives that are correctly classified. TNR is calculated by:(2)

The combinations of methods were compared in two ways. Firstly, the classification accuracies were compared, to determine which ones gave the highest accuracy. Then, the attributes selected by the FS techniques, were analysed using DAVID bioinformatics resource to identify the genes or proteins in each reduced dataset, from their microarray identifiers [32]. The genes and proteins included in the BioHEL reduced datasets were assessed by literature searches with PubMed, to determine any known relevance of the attributes to OA and any other disease or treatment classes included in the datasets.

## Results

### The RGIFE heuristic increased the classification accuracy achieved for both proteomics and transcriptomics data

By applying the RGIFE FS heuristic to the canine proteomics dataset, the classification accuracies achieved were considerably higher compared to the accuracy achieved without any feature selection (Table 2). This result is robust to choice of protein quantification (emPAI or ProteinProphet). In previous work [25] we showed that BioHEL performed favourably as compared with other classification techniques, best performing with ProteinProphet probabilities (classification accuracy of 73.9%), but out-performed by JRip with emPAI values (classification accuracy of 78.3%). Thus our new feature reduction method, with TPR and TNR of 96% and 99% respectively, has considerably outperformed all other methods for classification accuracy on this dataset.

**Table 2:** Classification accuracies achieved using BioHEL with and without also using the RGIFE heuristic.

Classifier	BioHEL, no feature reduction		RGIFE+BioHEL	
	
	TPR	TNR	TPR	TNR
ProteinProphet	0.74	0.90	0.91	0.97

emPAI	0.57	0.81	0.96	0.99

All five transcriptomics datasets were analysed using the seven ML methods and BioHEL+RGIFE (Table 3). For three of the five transcriptomics datasets, BioHEL without feature reduction gave a classification that was higher or equal to the other methods. For the other two datasets, GSE36700 and E-GEOD-29746, the best methods were IBk and SVM. The application of RGIFE increased the classification of all five datasets, except for E-GEOD-27290, which was already at 100%. After the feature reduction, only one dataset, E-GEOD-27946, was classified better using an alternative method, SVM.

**Table 3:** TPR and TNR achieved by BioHEL compared to the other best methods for the five transcriptomics datasets, using leave-one-out cross validation

	NaiveBayes		SVM		IBk		Jrip		J48		RandomForest		BioHEL		RGIFE+ BioHEL	
	**TPR**	**TNR**	**TPR**	**TNR**	**TPR**	**TNR**	**TPR**	**TNR**	**TPR**	**TNR**	**TPR**	**TNR**	**TPR**	**TNR**	**TPR**	**TNR**

**GSE36700**	0.24	0.67	0.28	0.72	0.84	0.97	0.48	0.66	0.24	0.62	0.44	0.83	0.76	0.91	0.96	0.98

**GSE3698**	0.58	0.73	0.39	0.60	0.67	0.79	0.67	0.78	0.75	0.84	0.54	0.72	0.73	0.83	1.00	1.00

**E-GEOD-12021**	0.84	0.91	0.39	0.61	0.71	0.83	0.58	0.73	0.58	0.74	0.77	0.87	0.87	0.93	0.97	0.98

**E-GEOD-29746**	0.48	0.63	0.48	0.68	0.65	0.79	0.61	0.76	0.42	0.59	0.55	0.71	0.77	0.87	0.84	0.90

**E-GEOD-27390**	1.00	1.00	1.00	1.00	0.95	0.95	0.90	0.89	0.90	0.91	0.84	0.86	1.00	1.00	1.00	1.00

### RGIFE compares favourably with other feature reduction methods

The comparison shown above could be construed as unfair, as it is comparing a classification using the attributes identified by a feature selection method with classifications using the whole dataset. Therefore, it is plausible that application of other feature selection methods could provide improvements comparable to the RGIFE technique. To test this, we have analysed both the proteomics and the transcriptomics datasets using all combinations of the FS and classification method methods. Detailed results are given in Supplementary Tables 1-7 (addition file 1).

For the proteomics dataset, RGIFE is the best feature reduction method, both with emPAI and ProteinProphet values. Using emPAI values (Supplementary Table 1, addition file 1), the highest accuracy was achieved by BioHEL for both FS and classification (TPR 96%; TNR 99%). This is compared to the highest achieved prior to FS, of 78.3%, using JRip, a rule-based classifier. Using ProteinProphet values (Supplementary Table 2, addition file 1), the accuracy rose from the highest accuracy of 73.9% using BioHEL to 95.7% after application of FS. The two FS and classification method combinations that gave this highest classification are: RGIFE with IBk (TPR 96%; TNR 99%) and Naïve Bayes for both FS and classification (TPR 96%; TNR 98%). The combination of RGIFE+BioHEL compares reasonably favourably with these scores (TPR 91%; TNR 97%).

The five transcriptomics datasets were also analysed using the different FS and classification method combinations, with mixed results. For GSE3698 (Supplementary Table 3, addition file 1), classification accuracies of 100% were achieved using RGIFE+BioHEL, as well as with a combination of SVM RFE FS with either SVM or IBk. For GSE36700 (Supplementary Table 4, addition file 1), SVM RFE FS again gave the highest accuracy of 100%, when used with NB, SVM or IBk. For this dataset, RGIFE misclassified only one of the 25 samples. Only one sample was also misclassified when RGIFE was applied to E-GEOD-12021 (Supplementary Table 5, addition file 1), and again SVM RFE combined with NB, SVM or IBk gave the highest accuracy of 100%. For dataset E-GEOD-27390 (Supplementary Table 6, addition file 1), a number of FS and classification methods resulted in classifications of 100% accuracy. This included the combination of RGIFE with either BioHEL or RF. The FS methods CFS and RF also resulted in classifications of 100% when used with various classification methods. Dataset E-GEOD-29746 (Supplementary Table 7, addition file 1) was generally less well classified by the various methods, with only two FS and classification method combinations resulting in classifications of 100% accuracy. These were SVM RFE with SVM and RF for FS with RF. Thus for these datasets, no single method stands out as being best.

For each combination of classifier and dataset, the best and worse methods were recorded, considering both the TPRs and TNRs. Tables 6 and 7 compare the six FS methods; they show that the method that most frequently resulted in the highest accuracy was SVM RFE, however it also frequently resulted in the lowest TPR and TNR as well, showing it to be very unstable. The comparison showed RGIFE to be the second best for the highest accuracy, and the second worst for the lowest accuracy, showing it to be the most stable across the datasets tested.

### Feature reduction identifies inflammation-associated proteins, which have been associated previously with cartilage matrix degradation

RGIFE identified ten proteins: six using the ProteinProphet quantification and five using emPAI values, with one protein (MMP-3) in common (Table 4). Six of these proteins found have been previously associated with cartilage inflammation and OA: MMP-3 [33], IL-8 [34], thrombospondin-1 [35], hyaluronan and proteoglycan link protein 1 [36], clusterin [37] and fetuin-A [38].

**Table 4:** The proteins included in the reduced datasets identified by RGIFE for the canine proteomics emPAI and ProteinProphet data.

Protein ID	Protein name	Identified from emPAI or ProteinProphet	Protein description	Known link to cartilage inflammation or OA
MMP-3	matrix-metalloproteinase 3	ProteinProphet and emPAI	MMP-3 is a proteolytic enzyme known to degrade components of the ECM, including collagens and cartilage proteoglycans [53].	Found to be down-regulated in late OA [33].

IL-8	interleukin-8	ProteinProphet	IL-8 is a chemotactic factor that attracts neutrophils, basophils, and T-cells, but not monocytes. It is also involved in neutrophil activation. It is released from several cell types in response to an inflammatory stimulus [54].	IL-8 is the major chemotactic factor released in response to proinflammatory cytokines in synovial tissues from RA and OA affected joints [34].

TSP1	thrombospondin-1	ProteinProphet	Adhesive glycoprotein that mediates cell-to-cell and cell-to-matrix interactions [55].	Levels of TSP1 are increased after the onset of OA [35]

APOE	apolipoprotein E	ProteinProphet	APOE mediates the binding, internalization, and catabolism of lipoprotein particles [56].	No known link.

HPLN1	hyaluronan and proteoglycan link protein 1	ProteinProphet	Stabilizes the aggregates of proteoglycan monomers with hyaluronic acid in the extracellular cartilage matrix [57].	HPLN1 has been associated with OA and osteophyte formation [36].

TPIS	triosephosphate isomerase	ProteinProphet	Catalyses the reaction D-glyceraldehyde 3-phosphate = glycerone phosphate [58].	No known link.

CLUS	clusterin	emPAI	A glycoprotein that functions as extracellular chaperone that prevents aggregation of non-native proteins, which is involved in many diverse biological functions [59].	Higher levels of clusterin have been observed in synovial fluid of advanced primary knee and hip OA patients [37].

FETUA	alpha-2-HS-glycoprotein/fetuin-A	emPAI	Promotes endocytosis, possesses opsonic properties and influences the mineral phase of bone [60].	FETUA levels have been found to decrease as the severity of knee OA increases [38].

POLG	Genome polyprotein	emPAI	Bacterial protein.	No known link.

ATPX	ATP synthase subunit b'	emPAI	Bacterial protein.	No known link.

### The reduced datasets generated from the transcriptomics datasets vary in size and utility

All the transcriptomics datasets were vastly reduced in the number of genes using BioHEL reduction. Table 5 shows the number of genes in each dataset both before and after application of RGIFE. The numbers of genes the datasets were reduced to varied: four out of five were between five and twenty-four genes (a reasonable number). However, E-GEOD-29746 gave 669 genes (out of the original 44397 genes), a much larger number.

**Table 5:** The number of genes present in each dataset before and after feature reduction with RGIFE

Dataset	Whole dataset No. of genes identifiers	Reduced datasets No. of genes identifiers
GSE36700	54675	24

GSE3698	17048	19

E-GEOD-12021	22284	5

E-GEOD-29746	44397	669

E-GEOD-27390	54675	14

The genes present in all the reduced subsets identified by RGIFE are listed in Supplementary Tables 8 – 11 (addition file 1), except for E-GEOD-29746, due to the large number of genes included. Of the genes included in the GSE36700 reduced dataset, two genes, RSAD2 and CXCL9, were found to be associated with RA, one of the disease groups included in the datasets [39,40]. In the GSE3698 reduced dataset, four genes are clearly relevant to the diseases analysed with this dataset. These genes are FN1, DDR2, MMP-9 and NOTCH3, all of which have been associated with either OA or RA [41-44]. Dataset E-GEOD-12021 was reduced to only five genes. Included in this small subset of genes was CXCL13, which has previously been suggested as a possible biomarker for RA [45]. None of the genes in the E-GEOD-27390 reduced dataset were found to be specifically related to OA or RA, the two disease classes included in the dataset. However, there were a number of genes whose functions are currently unknown.

## Discussion

### Comparison of classification accuracies achieved by feature selection methods

From the results shown, it can be seen that there is not one FS and classification method combination that is best for all datasets. For the proteomics data, the RGIFE+BioHEL combination compared very well with other methods. For all five of the transcriptomics datasets, at least one combination gave a classification accuracy of 100%. However, there was a lot of variation both in the accuracies of the classifications for each dataset, using the different methods, and the classifications performed by each method across the different datasets.

RGIFE gave the equal highest accuracies for two of the five transcriptomics datasets and was close to the highest accuracies for another two datasets. However, for dataset E-GEOD-29746 (the dataset where the heuristic was not very effective at identifying a very reduced set of features), other methods were better.

### Proteins identified from the proteomics dataset by RGIFE

Using the two different values for the proteins, emPAI and ProteinProphet, did result in different proteins being selected, however MMP-3 was included in both. This is because emPAI is a measure of relative protein quantitation and ProteinProphet gives a probability based on how likely it is a protein is present in a sample. Therefore, whilst these measures are related, they are not the same.

Included in the proteomics emPAI-reduced dataset are two bacterial proteins, Genome polyprotein (POLG) and ATP synthase subunit b' (ATPX). These proteins have fairly low Mascot scores, indicating a lack of confidence in this identification and so, based on this, it may be suitable to increase the Mascot score threshold to reduce the likelihood of false positives. Also, POLG was found in only three samples and ATPX was present in only one sample. Through analysis of the rules generated by BioHEL it is also clear that these proteins were used to distinguish between the control and carprofen-only treated samples, indicating that carprofen treatment has no detectable direct physiological effect.

Supplementary tables 12 and 13 (addition file 1) show the proteins selected by the other feature selection methods tested. All the proteins identified by RGIFE for the MS dataset, using ProteinProphet probabilities, were also identified by at least one of the other FS techniques tested. For the emPAI values dataset, two of the five proteins, CLUS & MMP-3, were identified by other FS methods. These proteins have previously been identified in OA [33,46]. For the dataset using emPAI values, other methods did also select bacterial proteins.

### BioHEL reduction method applied to transcriptomics datasets

The genes identified by RGIFE were also compared to the genes selected by the other FS techniques used. Supplemental tables 14-17 (addition file 1) list the genes identified by each method, for comparison with the genes included by RGIFE. For datasets E-GEOD-12021 and E-GEOD-27390 no genes selected by RGIFE were included in the lists of genes identified by the other FS methods. There was some overlap between the genes selected by the other methods, however only very few. Dataset GSE36700 had one gene, RSAD2, which was included in the reduced dataset of another FS method. RSAD2 has been found to be up-regulated in RA [39], which was a disease assessed by the GSE36700 investigation. Five of the genes selected by RGIFE for this dataset were also identified by other FS methods. When faced with such high dimensional datasets, the results show that the natural bias of each FS method produces very divergent results. Perhaps this is not surprising since all of them are heuristic.

The transcriptomics reduced-datasets were generally larger than those of the proteomics datasets. Quite likely this is due to proteomics datasets having far fewer features than transcriptomics ones, and the features present are less noisy [19].

## Conclusions

The RGIFE feature-reduction method has been shown to be suitable for the analysis of both transcriptomics and proteomics data. The classification accuracies achieved by this method in combination with the BioHEL rule-based machine learning method were better than other machine learning methods used (without FS) for all datasets and better or equal for the majority of datasets with feature selection also applied.

The feature reduction method resulted in the selection of a subset of genes for all datasets, some of which had clear links to the diseases related to the datasets. A number of genes were identified that may be suitable as possible biomarkers, however they require further individual analysis to determine their relevance and suitability.

Given that this is a generic supervised machine-learning technique, this method should also be suitable for analysis of other forms of complex data, including data from other omics areas, such as metabolomics and lipidomics, however testing in this area is still required.

Some cytokines and chemokines in the joint, such as IL8 (see table 6), have increased activity during OA, [47] which drive the increased production and secretion of matrix degrading enzymes such as matrix metalloproteinases (MMPs) (i.e. MMP-3, see table 7) that mediate the destruction of articular cartilage. Therefore, the feature-reduction methods used in this study identified biologically relevant proteins.

**Table 6:** Comparison of FS methods applied to both transcriptomics and proteomics, for each combination of classifier and dataset. The number of times each FS method resulted in the highest TPR and the lowest TPR are shown.

Method	No. of times the method results in the highest TPR	No. of times the method results in the lowest TPR
CFS	3	15

Chisquared	2	4

NaiveBayes - FS	6	6

Random Forest - FS	10	6

RGIFE	13	5

SVM RFE	16	13

**Table 7:** Comparison of FS methods applied to both transcriptomics and proteomics, for each combination of classifier and dataset. The number of times each FS method resulted in the highest TNR and the lowest TNR are shown

Method	No. of times the method results in the highest TNR	No. of times the method results in the lowest TNR
CFS	3	14

Chisquared	3	4

NaiveBayes - FS	6	7

Random Forest - FS	10	7

RGIFE	12	5

SVM RFE	15	12

In summary, to our knowledge bioinformatics feature-reduction tools have never been applied to ‘omics’ data in the area of rheumatology. However, as more and more investigators are applying ‘omics’ techniques to tissues and cells from arthritic joints, feature-reduction methods such as this are likely to make a significant contribution to basic and clinical research in this area, especially the stratification of patients based on data from molecular markers of joint inflammation. Despite the growing burden of arthritic diseases, many pharmaceutical companies have abandoned the development of disease modifying OA drugs (DMOADs) because OA is a heterogeneous disease with a variety of phenotypes and pathophysiological drivers. Identification of novel biomarkers and further validation of existing “experimental” markers are likely to facilitate OA drug development. Bioinformatic studies of currently available data from *in vitro* cartilage models, animal models and OA patients will consolidate existing knowledge of markers of disease progression and reveal new targets that may be invaluable for DMOAD development. The inevitable growth of public and private datasets derived from large cohort studies of joint inflammation will provide further opportunities for applying feature reduction techniques for biomarker identification and validation. This should provide a paradigm shift in the diagnosis and treatment of arthritis, facilitating new drug discovery and improving the range of effective therapy options for chronic joint diseases.

## Disclosures

The authors disclose no competing financial interests. A. Mobasheri is the coordinator of the **D-BOARD Consortium** funded by European Commission Framework 7 program (EU FP7; HEALTH.2012.2.4.5–2, project number **305815**, Novel Diagnostics and Biomarkers for Early Identification of Chronic Inflammatory Joint Diseases). J. Bacardit and C. Hodgman are participants in D-BOARD.

## Author Contributions

All of the authors made substantial contribution to the study, the organisation and the conduct of the study, to carrying out the study /including acquisition of study data and to analysis and interpretation of study data. Conceived and designed the study: JB, AM. Performed the experiments and the data analysis: ALS, JB. Drafted the manuscript: ALS, JB, AM, DJS, CH. Read, edited and approved the final manuscript: ALS, DJS, CH, DA, MHA, AM, JB. Contributed datasets: AM.

## Authors information

Jaume Bacardit and Ali Mobasheri are member of the following European Commission Consortia:

The D-BOARD European Consortium for Biomarker Discovery, The Universities of Surrey, Nottingham and Newcastle, United Kingdom; http://cordis.europa.eu/projects/rcn/105314_en.html

The APPROACH Consortium (Applied Public-Private Research enabling OsteoArthritis Clinical Headway) http://ec.europa.eu/research/health/medical-research/severe-chronicdiseases/projects/d-board_en.html (Website under construction)

## List of abbreviations

ATPX: ATP synthase subunit b'; CFS: Correlation-based Feature Selection; CLUS: Clusterin; CXCL9: Chemokine (C-X-C motif) ligand 9; DDR2: Discoidin domain receptor family, member 2; emPAI: Exponentially modified protein abundance index; FN1: Fibronectin 1; FS: Feature Selection; IBk: k-nearest neighbour; IFE: Iterative Feature Elimination; IL-1β: Interleukin 1 beta; ML: Machine Learning; MMP-3: Matrix metalloproteinase-3 (stromelysin-1); MMP-9: Matrix metalloproteinase-9; MS: Mass spectrometry; NB: Naïve Bayes; NOTCH3: Notch homolog 3; NSAID: non-steroidal anti-inflammatory drug; OA: Osteoarthritis; POLG: Genome polyprotein; RA: Rheumatoid Arthritis; RF: Random Forest; RGIFE: Rule-guided Iterative Feature Elimination; RSAD2: Radical S-adenosyl methionine domain containing 2; SVM: Support Vector Machines; SVM RFE: SVM-Recursive Feature Elimination; TNR: True Negative Rate; TPR: True Positive Rate.

## Supplementary Material

Additional file 1 Click here for file

## Declarations

Publication charges for this article have been funded by the Center of Excellence in Genomic Medicine Research (CEGMR), King Abdulaziz University, Jeddah, 21589, Kingdom of Saudi Arabia.

This article has been published as part of *BMC Genomics* Volume 16 Supplement 1, 2015: Selected articles from the 2nd International Genomic Medical Conference (IGMC 2013): Genomics. The full contents of the supplement are available online at http://www.biomedcentral.com/bmcgenomics/supplements/16/S1
