# Supplementary material for: A machine learning heuristic to identify biologically relevant and minimal biomarker panels from omics data
Source: BMC Genomics. 2015 Jan 15;16(Suppl 1):S2. doi: 10.1186/1471-2164-16-S1-S2 (PMC4315157; doi:10.1186/1471-2164-16-S1-S2)
Supplement: Additional file 1 [file 1471-2164-16-S1-S2-S1.docx]

**Supplementary information**

Supplementary figure 1. The graphs compare the accuracies resulting from the different combinations of feature selection and classification methods for the canine proteomics data (using both the emPAI and ProteinProphet datasets). The number of features used by each method is shown after the feature selection method on the horizontal axis.

Supplementary figure 2. The five graphs compare the different combinations of feature selection and classification methods for the five transcriptomics datasets, and the resulting true positive rates. The number of features used by each method is shown after the feature selection method on the horizontal axis.

Supplementary table 1. TPRs and TNRs achieved by difference combinations of feature selection and machine learning methods for the proteomics dataset, using emPAI values. The combinations of feature selection and machine learning methods that gave then highest classification accuracies were RGIFE+BioHEL for both. The columns are classification methods and the rows are feature selection methods.

|  | **NaiveBayes** | | **SVM** | | **IBk** | | **Jrip** | | **J48** | | **RandomForest** | | **BioHEL** | |
| --- | --- | --- | --- | --- | --- | --- | --- | --- | --- | --- | --- | --- | --- | --- |
|  | **TPR** | **TNR** | **TPR** | **TNR** | **TPR** | **TNR** | **TPR** | **TNR** | **TPR** | **TNR** | **TPR** | **TNR** | **TPR** | **TNR** |
| **CFS** | 0.74 | 0.90 | 0.57 | 0.83 | 0.83 | 0.94 | 0.83 | 0.93 | 0.83 | 0.94 | 0.83 | 0.94 | 0.74 | 0.90 |
| **RF** | 0.57 | 0.81 | 0.65 | 0.86 | 0.87 | 0.95 | 0.78 | 0.91 | 0.87 | 0.95 | 0.87 | 0.95 | 0.70 | 0.70 |
| **SVM RFE** | 0.83 | 0.94 | 0.61 | 0.85 | 0.83 | 0.94 | 0.48 | 0.76 | 0.65 | 0.87 | 0.78 | 0.92 | 0.78 | 0.92 |
| **Chisquared** | 0.65 | 0.88 | 0.52 | 0.81 | 0.74 | 0.90 | 0.70 | 0.87 | 0.83 | 0.94 | 0.87 | 0.95 | 0.78 | 0.92 |
| **NaiveBayes** | 0.87 | 0.95 | 0.52 | 0.77 | 0.74 | 0.90 | 0.65 | 0.86 | 0.83 | 0.94 | 0.74 | 0.90 | 0.74 | 0.90 |
| **RGIFE** | 0.87 | 0.95 | 0.74 | 0.89 | 0.87 | 0.95 | 0.57 | 0.85 | 0.87 | 0.95 | 0.78 | 0.92 | 0.96 | 0.99 |

Supplementary table 2. TPRs and TNRs achieved by difference combinations of feature selection and machine learning methods for the proteomics dataset, using ProteinProphet probabilities. The combinations of feature selection and machine learning methods that gave then highest classification accuracies were RGIFE combined with IBk and Naïve Bayes feature selection combined with Naïve Bayes classification method. The columns are classification methods and the rows are feature selection methods.

|  | **NaiveBayes** | | **SVM** | | **IBk** | | **Jrip** | | **J48** | | **RandomForest** | | **BioHEL** | |
| --- | --- | --- | --- | --- | --- | --- | --- | --- | --- | --- | --- | --- | --- | --- |
|  | **TPR** | **TNR** | **TPR** | **TNR** | **TPR** | **TNR** | **TPR** | **TNR** | **TPR** | **TNR** | **TPR** | **TNR** | **TPR** | **TNR** |
| **CFS** | 0.78 | 0.92 | 0.91 | 0.97 | 0.78 | 0.92 | 0.74 | 0.89 | 0.70 | 0.88 | 0.74 | 0.90 | 0.61 | 0.82 |
| **RF** | 0.91 | 0.97 | 0.83 | 0.94 | 0.87 | 0.96 | 0.52 | 0.76 | 0.70 | 0.88 | 0.83 | 0.94 | 0.57 | 0.82 |
| **SVM RFE** | 0.74 | 0.9 | 0.83 | 0.94 | 0.87 | 0.95 | 0.65 | 0.85 | 0.65 | 0.86 | 0.83 | 0.93 | 0.65 | 0.86 |
| **Chisquared** | 0.83 | 0.94 | 0.91 | 0.97 | 0.78 | 0.92 | 0.65 | 0.85 | 0.70 | 0.88 | 0.65 | 0.85 | 0.57 | 0.82 |
| **NaiveBayes** | 0.96 | 0.98 | 0.87 | 0.95 | 0.87 | 0.95 | 0.70 | 0.88 | 0.70 | 0.88 | 0.78 | 0.92 | 0.61 | 0.82 |
| **RGIFE** | 0.78 | 0.91 | 0.83 | 0.94 | 0.96 | 0.99 | 0.70 | 0.82 | 0.65 | 0.86 | 0.70 | 0.88 | 0.91 | 0.97 |

Supplementary table 3. TPRs and TNRs achieved by difference combinations of feature selection and machine learning methods for GSE3698. RGIFE+BioHEL gave the highest classification accuracy, along with SVM RFE combined with SVM and IBk. The columns are classification methods and the rows are feature selection methods.

|  | **NaiveBayes** | | **SVM** | | **IBk** | | **Jrip** | | **J48** | | **RandomForest** | | **BioHEL** | |
| --- | --- | --- | --- | --- | --- | --- | --- | --- | --- | --- | --- | --- | --- | --- |
|  | **TPR** | **TNR** | **TPR** | **TNR** | **TPR** | **TNR** | **TPR** | **TNR** | **TPR** | **TNR** | **TPR** | **TNR** | **TPR** | **TNR** |
| **CFS** | 0.77 | 0.86 | 0.88 | 0.93 | 0.69 | 0.81 | 0.58 | 0.70 | 0.73 | 0.84 | 0.77 | 0.85 | 0.73 | 0.82 |
| **RF** | 0.56 | 0.72 | 0.50 | 0.66 | 0.69 | 0.80 | 0.73 | 0.82 | 0.77 | 0.87 | 0.79 | 0.87 | 0.83 | 0.9 |
| **SVM RFE** | 0.96 | 0.97 | 1.00 | 1.00 | 1.00 | 1.00 | 0.63 | 0.76 | 0.69 | 0.81 | 0.85 | 0.91 | 0.83 | 0.9 |
| **Chisquared** | 0.81 | 0.85 | 0.77 | 0.85 | 0.83 | 0.89 | 0.63 | 0.75 | 0.71 | 0.81 | 0.79 | 0.87 | 0.81 | 0.88 |
| **NaiveBayes** | 0.94 | 0.96 | 0.83 | 0.90 | 0.77 | 0.88 | 0.67 | 0.79 | 0.88 | 0.93 | 0.81 | 0.88 | 0.79 | 0.86 |
| **RGIFE** | 0.90 | 0.93 | 0.94 | 0.96 | 0.92 | 0.95 | 0.79 | 0.86 | 0.75 | 0.84 | 0.85 | 0.91 | 1.00 | 1.00 |

**Supplementary table 4. TPRs and TNRs achieved by difference combinations of feature selection and machine learning methods for GSE36700. SVM RFE gave the highest classification accuracy, when combined with Naïve Bayes, SVM and IBk.** **The columns are classification methods and the rows are feature selection methods.**

|  | **NaiveBayes** | | **SVM** | | **IBk** | | **Jrip** | | **J48** | | **RandomForest** | | **BioHEL** | |
| --- | --- | --- | --- | --- | --- | --- | --- | --- | --- | --- | --- | --- | --- | --- |
|  | **TPR** | **TNR** | **TPR** | **TNR** | **TPR** | **TNR** | **TPR** | **TNR** | **TPR** | **TNR** | **TPR** | **TNR** | **TPR** | **TNR** |
| **CFS** | 0.60 | 0.84 | 0.68 | 0.89 | 0.60 | 0.86 | 0.36 | 0.67 | 0.40 | 0.74 | 0.76 | 0.94 | 0.64 | 0.86 |
| **RF** | 0.72 | 0.89 | 0.68 | 0.87 | 0.56 | 0.82 | 0.40 | 0.72 | 0.92 | 0.96 | 0.92 | 0.96 | 0.68 | 0.87 |
| **SVM RFE** | 1.00 | 1.00 | 1.00 | 1.00 | 1.00 | 1.00 | 0.76 | 0.91 | 0.68 | 0.90 | 0.88 | 0.96 | 0.72 | 0.89 |
| **Chisquared** | 0.76 | 0.92 | 0.88 | 0.97 | 0.84 | 0.96 | 0.68 | 0.86 | 0.80 | 0.95 | 0.88 | 0.96 | 0.68 | 0.87 |
| **NaiveBayes** | 0.88 | 0.96 | 0.60 | 0.82 | 0.76 | 0.91 | 0.68 | 0.88 | 0.80 | 0.94 | 0.88 | 0.97 | 0.72 | 0.89 |
| **RGIFE** | 0.84 | 0.98 | 0.84 | 0.95 | 0.96 | 0.98 | 0.52 | 0.78 | 0.52 | 0.81 | 0.76 | 0.91 | 0.96 | 0.98 |

Supplementary table 5. TPRs and TNRs achieved by difference combinations of feature selection and machine learning methods for E-GEOD-12021. SVM RFE gave the highest classification accuracy, when combined with Naïve Bayes, SVM and IBk. The columns are classification methods and the rows are feature selection methods.

|  | **NaiveBayes** | | **SVM** | | **IBk** | | **Jrip** | | **J48** | | **RandomForest** | | **BioHEL** | |
| --- | --- | --- | --- | --- | --- | --- | --- | --- | --- | --- | --- | --- | --- | --- |
|  | **TPR** | **TNR** | **TPR** | **TNR** | **TPR** | **TNR** | **TPR** | **TNR** | **TPR** | **TNR** | **TPR** | **TNR** | **TPR** | **TNR** |
| **CFS** | 0.90 | 0.95 | 0.94 | 0.97 | 0.74 | 0.85 | 0.68 | 0.79 | 0.87 | 0.93 | 0.94 | 0.96 | 0.74 | 0.74 |
| **RF** | 0.81 | 0.89 | 0.90 | 0.96 | 0.87 | 0.93 | 0.68 | 0.81 | 0.87 | 0.93 | 0.87 | 0.93 | 0.94 | 0.94 |
| **SVM RFE** | 1.00 | 1.00 | 1.00 | 1.00 | 1.00 | 1.00 | 0.65 | 0.78 | 0.61 | 0.77 | 0.84 | 0.92 | 0.77 | 0.88 |
| **Chisquared** | 0.84 | 0.90 | 0.84 | 0.92 | 0.87 | 0.94 | 0.65 | 0.77 | 0.67 | 0.79 | 0.81 | 0.88 | 0.77 | 0.88 |
| **NaiveBayes** | 0.97 | 0.98 | 0.87 | 0.92 | 0.90 | 0.94 | 0.81 | 0.88 | 0.74 | 0.85 | 0.74 | 0.84 | 0.81 | 0.91 |
| **RGIFE** | 0.84 | 0.91 | 0.90 | 0.95 | 0.84 | 0.91 | 0.77 | 0.87 | 0.71 | 0.83 | 0.77 | 0.88 | 0.97 | 0.98 |

Supplementary table 6. TPRs and TNRs achieved by difference combinations of feature selection and machine learning methods for E-GEOD-27390. RGIFE combined with either Random Forest or BioHEL have an accuracy of 100%. Chisquared, when combined with Naïve Bayes, SVM and IBk, and RF combined with Naïve Bayes, IBk or RF also gave an accuracy of 100%. The columns are classification methods and the rows are feature selection methods.

|  | **NaiveBayes** | | **SVM** | | **IBk** | | **Jrip** | | **J48** | | **RandomForest** | | **BioHEL** | |
| --- | --- | --- | --- | --- | --- | --- | --- | --- | --- | --- | --- | --- | --- | --- |
|  | **TPR** | **TNR** | **TPR** | **TNR** | **TPR** | **TNR** | **TPR** | **TNR** | **TPR** | **TNR** | **TPR** | **TNR** | **TPR** | **TNR** |
| **CFS** | 1.00 | 1.00 | 1.00 | 1.00 | 1.00 | 1.00 | 0.90 | 0.89 | 0.95 | 0.94 | 0.90 | 0.91 | 0.90 | 0.90 |
| **RF** | 1.00 | 1.00 | 0.90 | 0.88 | 1.00 | 1.00 | 0.95 | 0.94 | 0.95 | 0.94 | 1.00 | 1.00 | 0.95 | 0.95 |
| **SVM RFE** | 0.84 | 0.82 | 0.95 | 0.94 | 0.84 | 0.85 | 0.63 | 0.61 | 0.79 | 0.79 | 0.79 | 0.79 | 0.42 | 0.38 |
| **Chisquared** | 1.00 | 1.00 | 1.00 | 1.00 | 1.00 | 1.00 | 0.90 | 0.89 | 0.95 | 0.95 | 0.90 | 0.88 | 0.95 | 0.95 |
| **NaiveBayes** | 0.95 | 0.94 | 0.95 | 0.95 | 0.95 | 0.95 | 0.90 | 0.89 | 0.95 | 0.94 | 0.95 | 0.95 | 0.95 | 0.95 |
| **RGIFE** | 0.79 | 0.77 | 0.95 | 0.94 | 0.90 | 0.89 | 0.84 | 0.84 | 0.95 | 0.94 | 1.00 | 1.00 | 1.00 | 1.00 |

Supplementary table 7. TPRs and TNRs achieved by difference combinations of feature selection and machine learning methods for E-GEOD-29746. The best combination was Random Forest for both FS and classification. The columns are classification methods and the rows are feature selection methods.

|  | **NaiveBayes** | | **SVM** | | **IBk** | | **Jrip** | | **J48** | | **RandomForest** | | **BioHEL** | |
| --- | --- | --- | --- | --- | --- | --- | --- | --- | --- | --- | --- | --- | --- | --- |
|  | **TPR** | **TNR** | **TPR** | **TNR** | **TPR** | **TNR** | **TPR** | **TNR** | **TPR** | **TNR** | **TPR** | **TNR** | **TPR** | **TNR** |
| **CFS** | 0.68 | 0.83 | 0.81 | 0.89 | 0.71 | 0.82 | 0.42 | 0.59 | 0.74 | 0.86 | 0.77 | 0.87 | 0.61 | 0.78 |
| **RF** | 0.77 | 0.87 | 0.52 | 0.65 | 0.77 | 0.88 | 0.74 | 0.86 | 0.87 | 0.93 | 1.00 | 1.00 | 0.90 | 0.94 |
| **SVM RFE** | 0.94 | 0.97 | 1.00 | 1.00 | 0.97 | 0.98 | 0.61 | 0.76 | 0.84 | 0.92 | 0.81 | 0.91 | 0.77 | 0.87 |
| **Chisquared** | 0.68 | 0.80 | 0.71 | 0.83 | 0.87 | 0.93 | 0.68 | 0.82 | 0.81 | 0.90 | 0.94 | 0.97 | 0.87 | 0.93 |
| **NaiveBayes** | 0.94 | 0.96 | 0.77 | 0.87 | 0.84 | 0.90 | 0.87 | 0.93 | 0.94 | 0.97 | 0.94 | 0.96 | 0.90 | 0.94 |
| **RGIFE** | 0.61 | 0.78 | 0.87 | 0.93 | 0.84 | 0.91 | 0.48 | 0.65 | 0.71 | 0.86 | 0.71 | 0.84 | 0.84 | 0.90 |

Supplementary table 8. The genes in present in the GSE36700 dataset reduction with RGIFE after conversion of gene identifiers using DAVID.

| **Gene ID** | **Gene** | **Description and any known relevance to OA** |
| --- | --- | --- |
| IGKV3D-15 | Immunoglobulin kappa variable 3D-15 (gene/pseudogene) | Unknown. |
| LAMP5 | Lysosome-associated membrane glycoprotein 5 | A member of the LAMP family. |
| IFI6 | Interferon, alpha-inducible protein 6 | IFI6 has been reported to be involved in cell survival through the inhibition of apoptosis [[61](#_ENREF_61)]. |
| Fn3k | Fructosamine 3 kinase | Fn3k is involved in cell metabolism and is related to deglycation of fructoselysine and of glycated proteins [[62](#_ENREF_62)]. |
| LOC100126583 | Hypothetical LOC100126583 | Hypothetical protein |
| CYP2U1 | Cytochrome P450, family 2, subfamily U, polypeptide 1 | CYP2U1 is required for fatty acid signaling processes in both cerebellum and thymus [[63](#_ENREF_63)]. |
| DYDC1 | DPY30 domain containing 1 | DYDC1 is a protein found in the testis, which belongs to the dpy-30 family [[64](#_ENREF_64)]. |
| IGLC1 | Immunoglobulin lambda | Contains an immunoglobulin-like domain [[65](#_ENREF_65)]. |
| FHL1 | Four and a half LIM domains 1 | FHL1 is involved in muscle development. Found to be down-regulated after IL-1β treatment [[66](#_ENREF_66)]. |
| TP53BP2 | Tumour protein p53 binding protein, 2 | tp53bp2 is involved in the regulation of apoptosis and cell growth [[67](#_ENREF_67)]. |
| THSD7A | Thrombospondin, type I, domain containing 7A | THSD7A is known to promote endothelial cell migration and has been linked to osteoporosis [[68](#_ENREF_68)]. |
| DMRT3 | Doublesex and mab-3 related transcription factor 3 | Involved in embryonic development. |
| FAM30A | Putative uncharacterized protein KIAA0125 | Unknown. |
| RSAD2 | Radical S-adenosyl methionine domain containing 2 | RSAD2 is an IFN-inducible anti-viral protein, which is induced by human cytomegalovirus [[69](#_ENREF_69)]. RSAD2 has been found to be up-regulated in RA [[39](#_ENREF_39)]. |
| PLA2G2D | Phospholipase A2, group IID | PLA2G2D is an enzyme which catalyses the calcium-dependent hydrolysis of the 2-acyl groups in 3-sn-phosphoglycerides. It has been linked to cytokine mediated inflammation [[70](#_ENREF_70)]. |
| CXCL9 | Chemokine (C-X-C motif) ligand 9 | A cytokine which affects the growth, activation state and movement of cells involved in inflammation and the immune system [[71](#_ENREF_71)]. CLCX9 has been reported at higher levels in synovial tissue from RA patients [[40](#_ENREF_40)]. |
| IGHD | Ig delta chain C region | IgD is an antigen receptor on the surface of B-cells [[72](#_ENREF_72)]. |
| VSIG7 | V-set and immunoglobulin domain containing 7 | Unknown. |
| HOXB9 | Homeobox B9 | Involved in embryonic development [[73](#_ENREF_73)]. |
| DHX34 | DEAH (Asp-Glu-Ala-His) box polypeptide 34 | An ATP-binding RNA helicase involved in embryonic development [[74](#_ENREF_74)]. |
| TRPM2 | transient receptor potential cation channel, subfamily M, member 2 | TRPM2 is a voltage-independent cation channel mediating sodium and calcium ion influx in response to oxidative stress [[75](#_ENREF_75)]. |
| IGKJ5 | Immunoglobulin kappa joining 5 | Unknown. |
| 1557896_at | Unknown. | Unknown. |

Supplementary table 9. The genes present in the RGIFE-reduced GSE3698 dataset after conversion of gene identifiers using DAVID.

| **Gene ID** | **Gene** | **Description and any known relevance to OA** |
| --- | --- | --- |
| WBSCR5 | Williams-Beuren syndrome chromosome region 5/ Linker for activation of T-cells family member 2 | WBSCR5 is involved in FCER1-mediated signalling in mast cells [76]. |
| MGC71993 | Similar to DNA segment, Chr 11/ Ribonuclease kappa | MGC71993 is an endoribonuclease which cleaves phosphodiester bonds [[77](#_ENREF_77)]. |
| FN1 | Fibronectin 1 | Fibronectins are involved in various processes including cell motility, adhesion and maintenance of cell shape. Fibronectin is also involved in osteoblast compaction and mineralization [[41](#_ENREF_41)]. |
| FAM46A | Family with sequence similarity 46, member A | A member of the FAM46 family, which has been identified in ocular tissues [[78](#_ENREF_78)]. |
| LSAMP | Limbic system-associated membrane protein, Apolipoprotein L, 3 | LSAMP is involved in the mediation of neuronal growth and axon targeting [[79](#_ENREF_79)]. |
| SGPL1 | Sphingosine-1-phosphate lyase 1 | SGPL1 is known to cleave phosphorylated sphingoid bases into fatty aldehydes and phosphoethanolamine and elevates apoptosis [[80](#_ENREF_80)]. |
| STK24 | Serine/threonine kinase 24 | STK24 is a serine/threonine-protein kinase that promotes apoptosis in response to caspase activation and stress [[81](#_ENREF_81)]. |
| COL22A1 | Collagen, type XXII, alpha 1 | COL22A1 functions as a cell adhesion ligand for skin epithelial cells and fibroblasts [[82](#_ENREF_82)]. |
| CNGA1 | Cyclic nucleotide gated channel alpha 1 | The opening of the cation channel and thereby causing a depolarization of rod photoreceptors [[83](#_ENREF_83)]. |
| CD3D | CD3D antigen | CD3D is involved in T-cell maturation, through mediation of signal transduction [[84](#_ENREF_84)]. |
| PARP9 | Poly (ADP-ribose) polymerase family, member 9 | PARP9 is involved in PARP1-dependent DNA damage repair [[85](#_ENREF_85)]. |
| CLECSF6 | C-type lectin domain family 4, member A | CLECSF6 is thought to have a role in the regulation of immune reactivity and modulating dendritic cells (DC) differentiation and maturation [[86](#_ENREF_86)]. |
| DDR2 | Discoidin domain receptor family, member 2 | DDR2 is a cell surface receptor known to bind type II collagen and up-regulate MMP-13. MMP-13 digests type II collagen, which is key to OA [[42](#_ENREF_42)]. |
| MMP-9 | Matrix metalloproteinase 9 | The gelatinase MMP-9 is an enzyme involved in inflammatory diseases. Higher levels of MMP-9 have been identified in synovial fluids from RA and OA patients [[43](#_ENREF_43)]. |
| NOTCH3 | Notch homolog 3 | NOTCH3 is involved in regulation of cell fate determination by acting as a receptor for specific membrane bound ligands. NOTCH3 affects differentiation, proliferation and apoptosis of cells and has been linked to RA [[44](#_ENREF_44), [87](#_ENREF_87)]. |
| HEYL | Hairy/enhancer-of-split related with YRPW motif-like | HEYL acts as a downstream effector of Notch signalling which is thought to be involved in cardiac development [[88](#_ENREF_88)]. |
| S100A7 | S100 calcium binding protein A7 (psoriasin 1) | S100A7 is involved in calcium responsive signalling and has been found to be over expressed in inflammatory diseases [[89](#_ENREF_89)]. |
| CADPS2 | Ca2+-dependent activator protein for secretion 2 | Involved in large dense-core vesicle (LDCV)-regulated exocytosis; it acts as a calcium sensor in constitutive vesicle trafficking and secretion [[90](#_ENREF_90)]. |
| Hs.126945 (Unigene ID) | Transcribed locus | Unknown. |

Supplementary table 10. The genes present in the RGIFE-reduced E-GEOD-12021 dataset after conversion of gene identifiers using DAVID.

| **Gene ID** | **Gene** | **Description and any known relevance to OA** |
| --- | --- | --- |
| CXCL13 | Chemokine (C-X-C motif) ligand 13 | Chemotactic for B-lymphocytes but not for T-lymphocytes, monocytes and neutrophils. Suggested as a marker for RA. Elevated baseline CXCL13 levels were associated with increased rates of joint destruction [[45](#_ENREF_45)]. |
| UBD | Ubiquitin D | Ubiquitin-like protein modifier that can be covalently attached to target protein and subsequently leads to their degradation by the 26S proteasome. Increased expression of UBD found after treatment of human synovial fibroblasts isolated from patients with inflammatory arthritis with TNF-α [[91](#_ENREF_91)]. |
| TPD52 | Tumor protein D52-like 1 | The protein is reported to be involved in cell proliferation and calcium signalling. |
| LRC42 | Leucine rich repeat containing 42 | Belongs to the LRRC42 family. |
| RM18 | Mitochondrial ribosomal protein L18 | Together with thiosulfate sulfurtransferase (TST), acts as a mitochondrial import factor for the cytosolic 5S rRNA. |

Supplementary table 11. The genes present in the reduction by RGIFE of dataset E-GEOD-27390 after conversion of gene identifiers using DAVID.

| **Gene ID** | **Gene** | **Description and any known relevance to OA** |
| --- | --- | --- |
| PKNOX2 | PBX/knotted 1 homeobox 2 | PKNOX2 is a transcription factor involved in cell proliferation, differentiation and death [[92](#_ENREF_92)]. |
| CABP1 | Calcium binding protein 1 | CABP1 inhibits agonist-induced intracellular calcium signalling [[93](#_ENREF_93)]. |
| RDH10 | Retinol dehydrogenase 10 (all-trans) | RDH10 is a retinol dehydrogenase that converts all-trans-retinol to all-trans-retinal. It is required for limb, craniofacial, and organ development [[94](#_ENREF_94)]. |
| LOC283177 | Hypothetical protein LOC283177 | Hypothetical protein |
| FBXO36 | F-box protein 36 | Unknown. |
| LOC344595 | Hypothetical LOC344595 | Hypothetical protein |
| ATAD2 | ATPase family, AAA domain containing 2 | ATAD2 has been found to be Involved in the estrogen-induced cell proliferation and cell cycle progression of breast cancer cells [[95](#_ENREF_95)]. |
| PRPF18 | PRP18 pre-mRNA processing factor 18 homolog | PRPF18 is involved in pre-mRNA splicing [[96](#_ENREF_96)]. |
| HBS1L | HBS1-like | hbs1l is a member of the GTP-binding elongation factor family [[97](#_ENREF_97)]. |
| LOC100131262 | Hypothetical LOC100131262 | Hypothetical protein |
| SLC6A2 | Solute carrier family 6 (neurotransmitter transporter, noradrenalin), member 2 | SLC6A2 is an amine transporter, which stops the action of noradrenaline [[98](#_ENREF_98)]. |
| HMGB3P19 | High mobility group box 3 pseudogene 19 | Unknown. |
| 236174_at | Unknown. | Unknown. |
| 227509_x_at | Unknown. | Unknown. |

**Supplementary table 12. Proteins selected by feature selection methods when applied to the canine proteomics dataset with ProteinProphet probabilities. The proteins in bold were selected by more than one method.**

| **CFS** | **RF** | **SVM RFE** | **Chi squared** | **Naïve Bayes** | **RGIFE** |
| --- | --- | --- | --- | --- | --- |
| **MMP3** | COMP | CAPP | **MMP3** | LYSC1 | **MMP3** |
| **APOE** | **MMP3** | **TPIS** | **MGP** | **MMP3** | **IL8** |
| **SECA2** | CD37 | **APOE** | **IL8** | **SECA2** | **TSP1** |
| **MGP** | SHBG | **IL8** | **SECA2** | TRY2 | **HPLN1** |
| **TPIS** | **TPIS** | URE1 | **APOE** | **MGP** | **APOE** |
| **IL8** | NRDZ | **SECA2** | **TSP1** | **TPIS** | **TPIS** |
|  | PVRIG | **HPLN1** | **TPIS** | **IL8** |  |
|  | **IL8** | FETUA | K2C1 |  |  |
|  | MLX | PRO1 |  |  |  |
|  |  | **MMP3** |  |  |  |

Supplementary table 13. Proteins selected by feature selection methods when applied to the canine proteomics dataset with emPAI values. The proteins in bold were selected by more than one method.

| **CFS** | **RF** | **SVM RFE** | **Chi squared** | **Naïve Bayes** | **RGIFE** |
| --- | --- | --- | --- | --- | --- |
| **PGCA** | **PGCA** | **A1AT** | **MMP3** | **PGCA** | **CLUS** |
| **CLUS** | **CLUS** | VIM1 | **CLUS** | **CLUS** | **MMP3** |
| **MMP3** | K1C9 | **YM22** | **IL8** | K2C1 | FETUA |
| **IL8** | CATA | **IL8** | ENOA | TRY1 | POLG |
|  | **MMP3** | PURL | **MGP** | **MGP** | ATPX |
|  | ENOB | **ALBU** | VIME | **A1AT** |  |
|  |  | XYNA | SAA | **ALBU** |  |
|  |  | LEPA | **PGCA** | **YM22** |  |
|  |  | PRO1 | TPIS |  |  |
|  |  | CLUS | LUM |  |  |

Supplementary table 14. Genes selected by feature selection methods when applied to GSE3698. Where IDs could not be converted the array IDs are reported. The genes in bold were selected by more than one method.

| **CFS** | **RF** | **SVM RFE** | **Chisquared** | **NaiveBayes** | **RGIFE** |
| --- | --- | --- | --- | --- | --- |
| ITGBL1 | HIF1AN | MIG-6 | TNIP2 | DKFZP547N043 | WBSCR5 |
| CFLAR | KIAA1458 | LOC221442 | **SGPL1** | IMAGp998A10184 | MGC71993 |
| ZNF324 | **VAMP8** | ASS | DDIT4 | DKFZp434H2215 | FN1 |
| EMP3 | SMC1L2 | **SEMA6D** | MARCKS | C6orf80 | **FAM46A** |
| IMAGp998C02653 |  | C6orf170 | SLC25A5 | ZCWCC1 | LSAMP |
| NGFR |  | UPF3A | NUPL2 | **SEMA6D** | **SGPL1** |
| MAGEB3 |  | **MMP9** | **CD3D** | AICDA | STK24 |
| IMAGp998C143515 |  | OLFM3 | **VAMP8** |  | COL22A1 |
| **FAM46A** |  | ATP6V1F | CTSL |  | CNGA1 |
| PCBD |  | **NOTCH3** | RALB |  | **CD3D** |
|  |  |  |  |  | PARP9 |
|  |  |  |  |  | CLECSF6 |
|  |  |  |  |  | DDR2 |
|  |  |  |  |  | **MMP9** |
|  |  |  |  |  | **NOTCH3** |
|  |  |  |  |  | HEYL |
|  |  |  |  |  | S100A7 |
|  |  |  |  |  | CADPS2 |
|  |  |  |  |  | Hs.126945 |

**Supplementary table 15. Genes** **selected by feature selection methods when applied to GSE36700. Where IDs could not be converted the array IDs are reported. The gene in bold was selected by more than one method.**

| **CFS** | **RF** | **SVM RFE** | **Chisquared** | **NaiveBayes** | **RGIFE** |
| --- | --- | --- | --- | --- | --- |
| NFKBID | CORO6 | CHSY3 | CUL2 | GBP1 | IGKV3D-15 |
| 1553462_at | KIAA1826 | NBL1 | LAIR2 | TRIOBP | LAMP5 |
| CCDC34 | GCH1 | RASGRP1 | RAB8A | **RSAD2** | IFI6 |
| OGDH | DUSP18 | MGAT3 | MEF2C |  | Fn3k |
| CKAP2 |  | PGF | SFRS14 |  | LOC100126583 |
| TSC22D4 |  | FN1 | ITBP3 |  | CYP2U1 |
| CD80 |  | CXorf57 | RAP2A |  | DYDC1 |
| DYNLRB1 |  | BTBD1 | SCARA3 |  | IGLC1 |
| SLC11A1 |  | IFIT3 | COL12A1 |  | FHL1 |
| FOXP2 |  | SIPA1L3 | FAM110B |  | TP53BP2 |
|  |  |  |  |  | THSD7A |
|  |  |  |  |  | DMRT3 |
|  |  |  |  |  | FAM30A |
|  |  |  |  |  | **RSAD2** |
|  |  |  |  |  | PLA2G2D |
|  |  |  |  |  | CXCL9 |
|  |  |  |  |  | IGHD |
|  |  |  |  |  | VSIG7 |
|  |  |  |  |  | HOXB9 |
|  |  |  |  |  | DHX34 |
|  |  |  |  |  | TRPM2 |
|  |  |  |  |  | IGKJ5 |
|  |  |  |  |  | 1557896_at |

Supplementary table 16. Genes selected by feature selection methods when applied to E-GEOD-12021. Where IDs could not be converted the array IDs are reported. The genes in bold were selected by more than one method.

| **CFS** | **RF** | **SVM RFE** | **Chisquared** | **NaiveBayes** | **RGIFE** |
| --- | --- | --- | --- | --- | --- |
| PKNOX2 | DDX24 | SPTBN1 | **CDH1** | TMEM80 | CXCL13 |
| RPS13 | TNFAIP3 | ACY1 | PFKFB3 | HEXA | UBD |
| RPL9 | STK38 | USP46 | E2F3 | **GABARAPL1** | TPD52 |
| LRP1 | RPL38 | SKAP2 | ADAMDEC1 |  | LRC42 |
| MCL1 |  | RNF125 | **GABARAPL1** |  | RM18 |
| MAP4 |  | ADAM7 | 215373_x_at |  |  |
| DNAJA1 |  | CDC14B | 217679_x_at |  |  |
| SNX17 |  | CSNK2A1 | RAP2C |  |  |
| **CDH1** |  | CYorf15B | RNF34 |  |  |
| MBNL1 |  | MICALL1 | HAUS2 |  |  |

Supplementary table 17. Genes selected by feature selection methods when applied to E-GEOD-27390. Where IDs could not be converted the array IDs are reported. The genes in bold were selected by more than one method.

| **CFS** | **RF** | **SVM RFE** | **Chisquared** | **NaiveBayes** | **RGIFE** |
| --- | --- | --- | --- | --- | --- |
| **HNRNPC** | **ERH** | NBL1 | EIF1 | **HNRNPC** | PKNOX2 |
| SEPT2 |  | RASGRP1 | EIF1 |  | CABP1 |
| NARS |  | MGAT3 | SIGLEC7 |  | RDH10 |
| **ERH** |  | PGF | ORC6L |  | LOC283177 |
| CLTC |  | FN1 | NSD1 |  | FBXO36 |
| **CALM1** |  | CXorf57 | 236898_at |  | LOC344595 |
| DDX24 |  | BTBD1 | 240616_at |  | ATAD2 |
| DYNLL1 |  | SIPA1L3 | **CALM1** |  | PRPF18 |
| PTP4A1 |  | IFIT3 | **CALM1** |  | HBS1L |
| BTG1 |  | CHSY3 | 1558801_at |  | LOC100131262 |
|  |  |  |  |  | SLC6A2 |
|  |  |  |  |  | HMGB3P19 |
|  |  |  |  |  | 236174_at |
|  |  |  |  |  | 227509_x_at |
